# High-Temperature Sensor Based on Fabry-Perot Interferometer in Microfiber Tip

**DOI:** 10.3390/s18010202

**Published:** 2018-01-12

**Authors:** Zhenshi Chen, Songsong Xiong, Shecheng Gao, Hui Zhang, Lei Wan, Xincheng Huang, Bingsen Huang, Yuanhua Feng, Weiping Liu, Zhaohui Li

**Affiliations:** 1 Guangdong Provincial Key Laboratory of Optical Fiber Sensing and Communications, Institute of Photonics Technology, Jinan University, Guangzhou 510632, China; zhenshichan@gmail.com; 2 Wuhan National Laboratory for Optoelectronics, Huazhong University of Science and Technology, Wuhan 430074, China; 3 Department of Electronic Engineering, College of Information Science and Techonology, Jinan University, Guangzhou 510632, China; gaosc825@163.com (S.G.); xchuang83@163.com (X.H.); huangbingsen@stu2016.jnu.edu.cn (B.H.); favinfeng@163.com (Y.F.); wpl@jnu.edu.cn (W.L.); 4 School of Physics and Optoelectronic Engineering, Guangdong University of Technology, Guangzhou 510006, China; zh999em@163.com; 5 Institute of Optoelectronic Material and Technology, South China Normal University, Guangzhou 510631, China; wallen-0407@163.com; 6 State Key Laboratory of Optoelectronic Materials and Technologies and School of Electronics and Information Technology, Sun Yat-sen University, Guangzhou 510275, China; tlzh88@jnu.edu.cn

**Keywords:** optical fiber sensor, temperature, FPI

## Abstract

A miniaturized tip Fabry-Perot interferometer (tip-FPI) is proposed for high-temperature sensing. It is simply fabricated for the first time by splicing a short length of microfiber (MF) to the cleaved end of a standard single mode fiber (SMF) with precise control of the relative cross section position. Such a MF acts as a Fabry-Perot (FP) cavity and serves as a tip sensor. A change in temperature modifies the length and refractive index of the FP cavity, and then a corresponding change in the reflected interference spectrum can be observed. High temperatures of up to 1000 °C are measured in the experiments, and a high sensitivity of 13.6 pm/°C is achieved. This compact sensor, with tip diameter and length both of tens of microns, is suitable for localized detection, especially in harsh environments.

## 1. Introduction

The research on reliable fiber-based high-temperature sensors that can operate near 1000 °C is a hot topic within the sensor community. These sensors have applications in many fields, such as combustors, metal melting furnaces, nuclear reactors, and so on [1,2]. Various fiber-based sensors have been demonstrated for temperature measurements up to 1000 °C with fused silica fibers, and even higher than 1500 °C with sapphire fibers [3]. Among these, different kinds of optical structures have been proposed to fabricate temperature sensors, such as Mach-Zehnder interferometers [4,5], Michelson interferometers [6,7], long-period gratings [8,9,10], regenerated fiber Bragg gratings [2], etc. Meanwhile, Fabry-Perot interferometers (FPI) have attracted continued attention for their simple configuration, compactness and good performance in high-temperature environments [1]. FPIs include intrinsic FPIs, in which the resonant cavities are fibers, and extrinsic FPIs, in which an air gap or fluids in the gaps serve as the cavities. The main objective is to fabricate two in-fiber reflectors for intrinsic FPIs. To achieve this, different kinds of structures have been developed in recent years [3,11,12,13,14]. Currently, most FPIs are manufactured by fusion splicing different types of optical components, such as photonic crystal fibers [15,16,17,18,19], crystalline silicon [20,21] and hollow-core silica tubes [14]. However, these specialized and expensive components would significantly increase the cost of the devices. Another method for fabricating FPIs is micromachining on the fiber with a high-power femtosecond laser [6,13], which increases both the production cost and fabrication complexity. The chemical etching method [22,23] is also commonly used to fabricate the fiber sensor. However, this approach would increase the fabrication steps and time. Additionally, some chemical reagents are harmful to health and the environment. In addition, many devices have relatively large sizes, of the order of millimeters or centimeters, which is not suitable for localized detection in some special applications.

In this paper, a tip-FPI based on a short section of microfiber (MF) is proposed, which is easy to fabricate. The MF is prepared by tapering SMF to around 30 μm in diameter and 60 μm in length, or even shorter. Therefore, it is tiny enough to be used for localized detection. The tip-FPI sensor is based on a microfiber Fabry-Perot cavity obtained by splicing a short section of MF to the cleaved end of a standard SMF. Here, the precise control over the relative cross section position is the key to forming the FPI, and this affects the total properties of the FPI, such as the contrast ratio of the interference fringe. Temperature responses in the range of 25–1000 °C were studied experimentally. Theoretical analysis was also carried out, and the results suggest the capability of our developed sensor for detecting high temperatures. The dips of the interference fringe display a red shift when the surrounding temperature increases. The sensitivity reaches 13.6 pm/°C, which is very close to the result of the numerical calculation.

## 2. Structure and Theory

**Figure 1:**
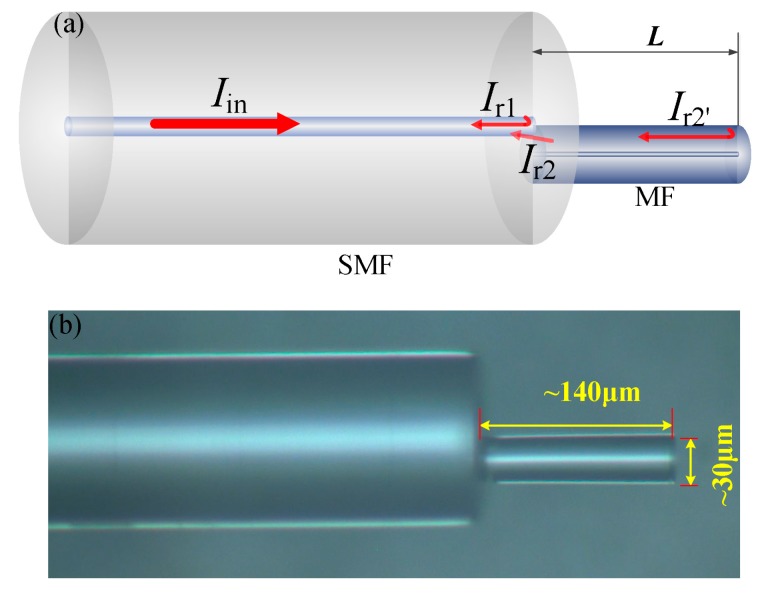
(**a**) Schematic structure of the tip-FPI; (**b**) top view of microscope of a tip-FPI sample.

The structure of the tip-FPI is schematically shown in Figure 1a. A portion of the incident light is reflected back to the core (*Ι*_r1_) when the light is launched into the SMF, due to the reflection from the SMF-air interface. The rest of the incident light is coupled to the MF and reflected back at the end of the MF (*Ι*_r2′_), due to the Fresnel reflection from the MF-air interface. At the junction, a part of the light (*Ι*_r2_) is coupled back into the guided core mode of the SMF. As a result, two beams of light (*I*_r1_ and *Ι*_r2_) interfere. At the same time, there is a part of *Ι*_r2′_ that is repeatedly reflected and coupled back into the SMF core. However, this part of energy is much lower than the first reflection. Therefore, only the first reflection process at the MF-air interface is considered in this paper. The top view of the fabricated device captured by optical microscope is shown in Figure 1b.

The cavity of the tip-FPI is MF, and the intensity of the interference light can be modeled simply by using the two-beam optical interference equation *Ι*_re_ = *Ι*_r1_ + *Ι*_r2_ + 2(*Ι*_r1_*Ι*_r2_)^1/2^cos(4π*n*_MF_*L*/*λ* + *φ*_0_) [24], where *Ι*_re_ is the intensity of the interference signal, *Ι*_r1_ and *Ι*_r2_ are the reflection intensities from the two interfaces, respectively. *n*_MF_ is the refractive index (RI) of the MF, *L* is the length of MF, and *λ* is the free-space wavelength. When the phase difference Δ*δ* = 4*πn*_MF_*L*/*λ* + *φ*_0_ = (2*m* + 1)π, *m* = 0, 1, 2,…, the interference minima occur at

(1) $$\lambda_{m}=\frac{4\pi n_{\mathrm{MF}}L}{\left(\left(2m+1\right)\pi -\varphi_{0}\right)}$$

where *λ*_m_ refers to the central wavelength of the mth order interference dip. The contrast of the interference fringe is determined by the intensities of the two beams of light (*Ι*_r1_ and *Ι*_r2_). The closer the intensity of the two beams, the higher the contrast ratio will be. It reaches its maximum when the two intensities are equal, which can be controlled by the adjustment of the relative cross-sectional position. The spectral fringe spacing between adjacent interference notches, namely, the free spectral range (FSR), can be derived and expressed as [25] *FSR* = *λ*_m_^2^/2*n*_MF_*L*. The *n*_MF_ and the *L* will change according to the variation of the environment, leading to changes in the interference spectrum. The change of a particular parameter could be determined according to the shift of the interference dip *λ*_m_. The variations of the refractive index and length of cavity can be denoted as Δ*n* and Δ*L*, respectively. Then, the wavelength shift can be expressed as

(2) $$\Delta \lambda_{m}=\lambda_{m}(\frac{\Delta n}{n_{\mathrm{MF}}}+\frac{\Delta L}{L})$$

When the temperature changes, Equation (2) is differentiated by temperature (T):

(3) $$\frac{d\lambda_{m}}{dT}=\lambda_{m}(\frac{1}{n_{\mathrm{MF}}}\frac{dn_{\mathrm{MF}}}{dT}+\frac{1}{L}\frac{dL}{dT})=\lambda_{m}(\delta +\alpha )$$

where *δ* = 8.3 × 10^−6^ °C^−1^ and *α* = 0.55 × 10^−6^ °C^−1^ [4] are the thermos-optic coefficient and thermal expansion coefficient of silica, respectively.

## 3. Experiment and Discussion

The fabrication procedures of the tip-FPI includes four steps [26]: (a) tapering SMF; (b) cleaving MF; (c) splicing the cleaved MF onto a cleaved SMF with optimized relative cross-sectional position; and (d) cleaving the MF to a designed length. Firstly, a standard SMF (SMF-28e, Corning, NY, USA) is tapered by using the flame-brushing technique [27]. The MF diameter can be controlled by adjusting the elongation speed and travel distance. Here, we tapered the SMFs with taper waist diameter and waist length of 30 μm and 15 mm respectively. Then, the tapered fiber was cleaved into two segments at its waist position with a smoothly cleaved end. In the following step, the pigtail of the cleaved tapered fiber(MF) was spliced onto one cleaved SMF at optimized relative cross-sectional position [28,29] by using a commercial fusion splicing machine (S178 A2, FURUKAWA, Kyoto, Japan) in manual operation mode. The MF was offset by about 4 μm from the center of the SMF core. It should be noted that the splicing position has more influence on the contrast of the interference fringe, but less on the sensing properties, as the sensitivity is determined by the thermos-optic coefficient and thermal expansion coefficient of silica. In order to avoid obvious deformation of the splicing interface, here the arc power and duration time were set to 110 and 100 ms, respectively, to fabricate an optimized diameter of 30 μm MF tip-FPI. Too large a diameter could cause heavy losses due to the reduction in the energy density of the reflected light. It is hard to detect the interference spectrum if the loss is too great. While too small a diameter would significantly increase fabrication complexity, both in cleaving and splicing. Finally, the MF was cleaved with the designed length, and the tip-FPI was fabricated, as shown in Figure 1.

**Figure 2:**
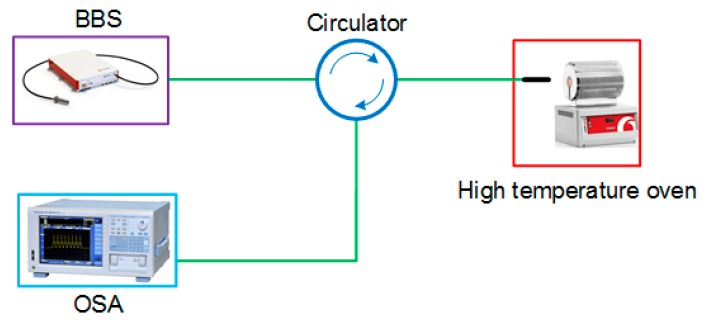
Schematic experimental setup of the tip-FPI sensing system.

**Figure 3:**
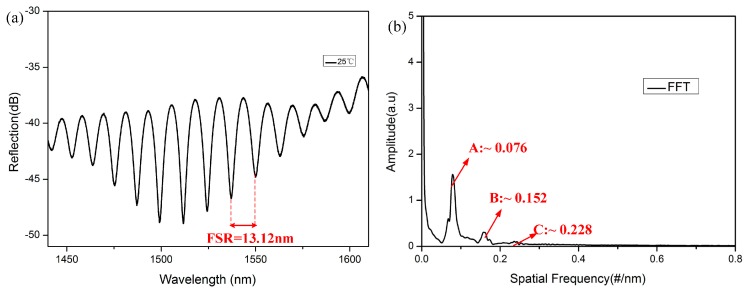
(**a**) Reflection spectrum of the device (*L* = 60 μm); (**b**) the FFT.

The experimental setup, as shown in Figure 2, consisted of a broadband optical source (Fianium, NKT Photonics, Southampton, UK), an optical fiber circulator, and an optical spectrum analyzer (AQ6370, YOKOGAWA, Kanazawa, Japan) with a resolution of 0.02 nm, which was used to measure the reflection spectral response of the tip-FPI. The tip-FPI was placed horizontally in the high-temperature oven (Carbolite Gero, Sheffield, UK). The reflection spectrum of the tip-FPI with a length of 60 μm is shown in Figure 3a. A fast Fourier transform (FFT) was performed to provide a deep insight into the frequency components, as shown in Figure 3b. Multiple peaks indicate multiple reflections from the MF-air interface. It is clear to see that the power of the reflected light was mainly concentrated in the lowest frequency component (for example A), and that of the high-frequency ones (for example B and C) quickly decrease with the increase of frequency. Obviously, the power at the lowest frequency component, mainly resulting from the first reflection, is dominant, and the high-frequency ones induced by multiple reflections are very small, such that they can be neglected for the purposes of this experiment.

**Figure 4:**
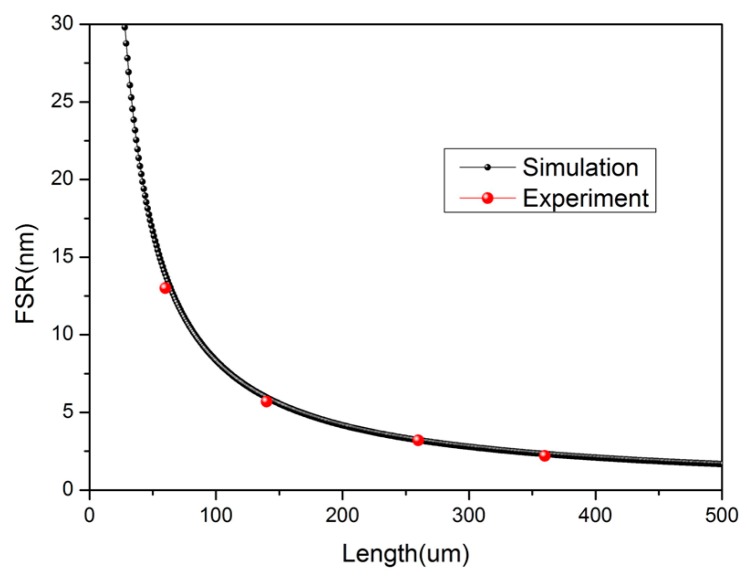
The relationship between *FSR* and *L* at the wavelength of 1550 nm.

From the analysis above, we can see that the *FSR* is related to the *n*_MF_ and *L* of the cavity. Considering the material of the MF is almost the same as the SMF, we took the value of 1.44 as the RI of a MF at 1550 nm. The calculated relationship between the *FSR* and *L* is shown in Figure 4. Observing the experimental spectrum in Figure 3, the value of the *FSR* is 13.12 nm. This implies that the *L* is 63.6 μm, which is very close to the direct measurement result of 60 μm obtained from the microscope image. Some other experimental results are also consistent with the theoretical results, and are shown as red balls in Figure 4.

**Figure 5:**
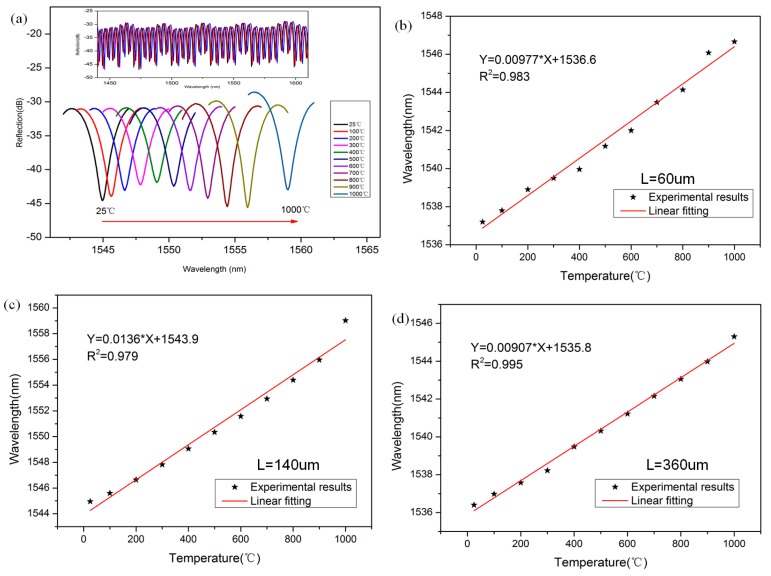
(**a**) Shift of reflection spectrum with temperature (*L* = 140 μm); the wavelength-temperature relationship and their linear fitting (**b**) *L* = 60 μm; (**c**) *L* = 140 μm and (**d**) *L* = 360 μm.

Figure 5a shows the reflection spectra of the tip-FPI when the atmosphere temperature was changed from 25 to 1000 °C. When the temperature increases, the dip moves to the long wavelength region. This result can be predicted by Equation (2), because all the values on the right side are positive. Tip-FPIs with different cavity lengths (60 μm, 140 μm and 360 μm) were fabricated, and the relationships between the temperature and dip wavelength were experimentally investigated, as shown in Figure 5b–d. The sensitivity of the tip-FPI-based temperature sensor can be estimated by fitting the slope of the experimental data. From the linear analysis, we can tell the sensitivity of the different lengths are 9.77 pm/°C, 13.6 pm/°C, and 9.07 pm/°C, with excellent linear properties, and the *R*^2^ values are 0.983, 0.979, and 0.995, respectively. These results are consistent with 13.72 pm/°C, which is deduced from Equation (3) at 1550 nm.

## 4. Conclusions

In this work, a compact tip-FPI temperature sensor was developed. The miniaturized sensor with excellent linearity can be simply fabricated, because it is only necessary to splice MF to the end of a SMF to construct an all fiber tip sensor. The tiny size of the sensor makes it very suitable for localized detection. When the temperature was increased to 1000 °C, the excellent sensing performance could also be obtained. A temperature sensitivity of 13.6 pm/°C was achieved in the range of 25–1000 °C. The experimental results are consistent with the theoretical analysis of reflection mode of the tip-FPI. The miniaturized sensor proposed in this paper is suitable for high-temperature sensing in harsh environments, especially in cases where the detection space is extremely small.
